# The Psychoanalytic Concept of *Jouissance* and the Kindling Hypothesis

**DOI:** 10.3389/fpsyg.2017.01593

**Published:** 2017-09-21

**Authors:** Yorgos Dimitriadis

**Affiliations:** Research Center for Psychoanalysis and Society, EA3522, UFR Psychoanalytic Studies, University Paris-Diderot Sorbonne-Cité Paris, France

**Keywords:** psychoanalysis, freud, lacan, kindling, sensitiztion, conditioning, psychosomatic disorders, brain

## Abstract

This article aims to define the conceptual field of *jouissance* in Lacanian theory, and put forth the hypothesis of a relationship between certain neurophysiological mechanisms and specific clinical phenomena where *jouissance* is “kindled” and outside the control of the symbolic process. First, the author briefly introduces Lacan's notion of *jouissance* and the way it draws on Freud's theorization, and describes the preliminary stages of this conceptual field in Lacan's work. Then, the *jouissance* related to two other concepts: repetition, with its Freudian and Lacanian nuances, as well as the—exclusively—Lacanian concept of the object *petit a*. Lacan's later conceptualization of language as *jouissance* (the notion of *lalangue*) is then discussed in relation to Freud's early ideas (“Letter 52”) on the different kinds of inscriptions that help form the mental apparatus. Finally, the author tries to formulate a hypothesis regarding specific neurophysiological mechanisms, based on clinical situations where *jouissance* becomes “kindled” and escapes the control of the symbolic processes through the neurophysiological mechanisms of conditioning, “kindling-sensitization” and “excitotoxicity.” In these cases, *jouissance* can have a destructive effect on the body and can affect, among others organs, the brain—a process the author has previously described heuristically as the “psychosomatic diseases of the brain.” This would be a special mechanism of automatism that would be triggered under the specific conditions of the fragility of the signifying chain (foreclosure of the Name-of-the-Father or solidification of the signifying chain) in combination with biological factors, including genetic factors. In this process, signifiers are reduced to signals, which in turn may be reduced to stimuli, with a tendency toward self-perpetuation, while affects are reduced to emotions and moods. Thus, conditioning and kindling-sensitization could also be understood in terms of a “semiotic reduction.” Can we therefore consider that certain phenomena of automatism and certain deficits (delusional moods, schizophrenic apathy, etc.) could be seen as psychosomatic disorders of the brain? The phenomena in question might also serve—albeit at random—as a kind of shield to mitigate excessive *jouissance*.

Jacques Lacan, in his 1969 seminar *The Other Side of Psychoanalysis* (Lacan, 1969–1970, session of 11 February 1970), said that if analysis had one task to complete, it was to create a new field of energetics, the field of *jouissance*, which would require other structures than those of physics. He also expressed regret that this field would not be called, as he would have wished, the “Lacanian field,” because with the little time he had left, he would not be able to even sketch out its basics. In the course of his teaching, Lacan designated seven versions of *jouissance*: of the Thing, of Being, of the Other (as subjective and objective genitive), of the body image, of the phallic image, sexual *jouissance*, and the *jouissance* of life. Things are quite different for Freud, who instead described different situations potentially referring to *jouissance* and including joy, pleasure, extreme pleasure, ecstasy, beatitude, sexual pleasure related to sexual satisfaction, the libido, and preliminary sexual excitement. The list already gives us an idea that *jouissance* is not pleasure, but rather, as Lacan announced in his lecture on *Psychoanalysis and Medicine*, pleasure is “what necessarily stops us at a certain point, at a respectful distance from *jouissance*” (Lacan, 1967, p. 46). In the same lecture, Lacan says that *jouissance* is “always of the order of tension, of forcing, of expenditure, even of exploit. *Jouissance* is undoubtedly there at the point where pain begins to appear” (Ibid).

## Preliminaries and the birth of the notion of *jouissance*

We will trace the trajectory of the concept of *jouissance* in Lacan, which not only has the status of the session of 5 March 1958 in his seminar *The Formations of the Unconscious* (Lacan, 1957–1958), where the term *jouissance* is introduced and contrasted with the notions of desire and the signifier. Two sessions later, on 25 March, Lacan says: “What we find at the basis of the analytical exploration of desire is masochism—the subject grasps himself as suffering; he grasps his existence of a living being as a signifier, i.e., as subject to desire,” and later: “The subject does not simply satisfy desire, he enjoys desiring (*jouit de désirer*) and this is an essential dimension of his *jouissance*.” Lacan will examine, while gradually revising it, this antithesis between *jouissance* and desire (but also between *jouissance* and the signifier) throughout the different stages of his teaching from 1957 to 1975, especially in his seminar R.S.I. Miller (1999) calls the first of the six consecutive examples of *jouissance* he finds in Lacan's work the “imaginarization” of *jouissance*, because the latter appears, he argues, as what resists symbolic elaboration, whether it is *acting out*, temporary perversions (e.g., in Seminar IV *On the Object-relation*), or the figure of the ferocious Superego. Following Miller's argument, we could say that, at this stage, Lacan's thinking about *jouissance*—which has not yet been named as such^1^—remains linked to the “beyond of the symbolic elaboration, on the broken horizon of which appears the fantasmatic products of *jouissance*.”

Aside from these preliminaries, the seminar where Lacan first develops the concept of *jouissance* is Seminar VII, *The Ethics of Psychoanalysis* (Lacan, 1959–1960a,b). Here he approaches *jouissance* through the prism of the impossible, linked to the notion of the Freudian Thing (Freud, 1950), *das Ding*, the subject's absolute Other. This is a “Real” as the subject's intimate exteriority, in other words, as what is most external yet closest to him. Likewise, the Thing is both the essence of Evil and the source of sublimation. The forbidden mother appears in this seminar as the incarnation of this absolute Other, i.e., of the Thing. Beyond the prohibition lies an impossibility—of the Real—announcing the divorce between *jouissance* and the signifier. The Thing is what cannot be found, due to not just prohibition but principal impossibility. The Oedipal prohibition therefore becomes a “mythical” version of this primordial impossibility. The *jouissance* of the Thing is impossible, hence Lacan's initial definition of *jouissance* as “the *jouissance* of the Thing as impossible.” In the same seminar, Lacan tries to define the notion of *jouissance* as the satisfaction of the drive—rather than need—a definition that he later never repeats. The aim of the drive is not satisfaction but, to the contrary, the failure of satisfaction, which restarts its circuit. Consequently, if *jouissance* is to be the satisfaction of the drive, it is only insofar as any drive is ultimately the death drive, in other words, insofar as the drive can run *jouissance* through the chain of signifiers, thus historicizing the subject and, by the same token, bringing *jouissance* out of the exclusive circuit of the living.

## The relation of *jouissance* to the object *petit a* and to repetition

**Figure 1:**
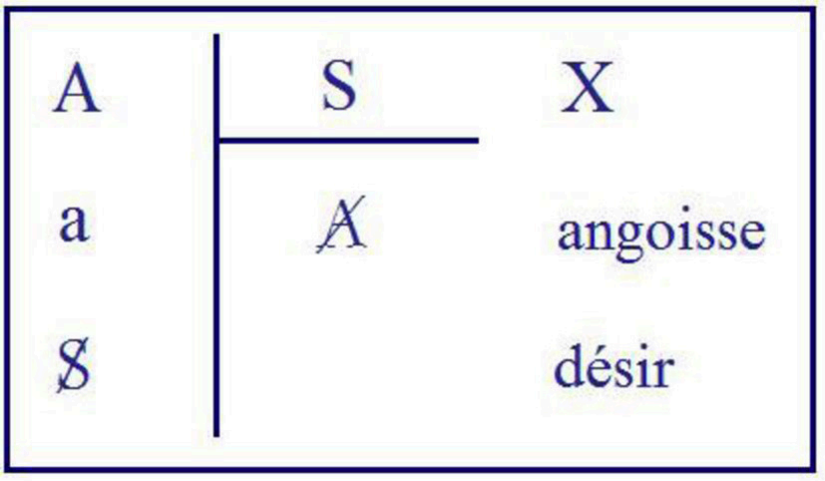
Second schema of subject's division, J. Lacan, seminar Anxiety.

In his seminar on *Anxiety* (Lacan, 1962–1963), during the session of 3 July 1963, Lacan says that for us, *jouissance* is not by nature promised to desire and that desire can only strive toward it. In the same seminar he speaks about *jouissance* via the operation of “subjective division” (Figure 1). In this division, the link between the signifiers and the desire of the Other is established (or not) through a process of subjectification, with anxiety as the intermediate level. The word “division” concerns the bar dividing A, the big Other, by the subject, who is inscribed as a quotient. How many times can the S fit into the A? The first stage of this division is the stage of mythic *jouissance* (“*jouissance* of Being,” marked as X in the schema of Figure 1) of the first undivided subject; this first relationship with A creates the liaison with the first signifier of the “desire of the Other.” This raises the question of “what the Other wants from me” in saying this or that to me, a question that produces anxiety—hence Lacan calls this second logical moment the “moment of anxiety.” It produces the inscription of *jouissance* as the object *petit a*, which is the remainder, the residue of this division. It is the subject who appears as *petit a* for the Other, who wants something from him and who is thus lacking (barred). The result of this division is the barred Other, who consequently appears as lacking. The subject, after having moved through the position of the *petit a* for the Other, who is lacking, divides, in the rest of the process, the *petit a* by the S, and at the same time is himself divided between the first signifier and the remaining signifiers coming from the Other. This is the logical moment of division, which creates the possible passage—via anxiety, as we have seen—from the subject's *jouissance* to his desire. The object *petit a* is thus the cause of this desire. In the seminar, Lacan announces that “only love allows *jouissance* to condescend to desire” (p. 179), referring to the fact that the coincidence of desire and love, however contingent—through love—is not an indispensable condition.

In the following seminar, *The* Four *Fundamental Concepts of Psychoanalysis* (Lacan, 1964), Lacan further elaborates the link between the *petit a* as an element of *jouissance* and the massive *jouissance* of the Thing it replaces. The *petit a* is, on the one hand, an essential figure of the Thing, but, on the other hand, it is linked to the Other as the locus of signifiers. Its role is to mediate between the Thing and the Other. The object *petit a* is an element and from this perspective resembles the signifier, which is also an element, namely because it is discontinuous. However, at the same time it has something to do with the essence, just like the Thing, in other words with the first bodily excitations and especially the body's orifices. Contrary to the Thing, the excitations of the object *petit a* have been subject to the desire of the Other, the Other who is lacking, as we have seen. This is because something in the structure of the body—in the functioning of the drives—is isomorphic to the structure of the unconscious. In this seminar, Lacan describes the unconscious as a dynamic system, rather than as a static series of repeating signifiers: it becomes a kind of edge that opens and closes, like an erogenous zone, like the mouth, and the anus. The lack of the signifier is articulated around the lack of the body. However, the latter is not the result of the signifier, rather it is natural and primary. *Jouissance* remains untamed as the *jouissance* of the raw body, of the living organism, yet it is also likely to become inscribed in the system of the signifiers of the unconscious, albeit never completely. In this seminar, *jouissance* is therefore not defined only negatively, as what is not assimilated by the Symbolic (the way we saw it in the seminar on *Ethics*). The link between *jouissance* and the signifiers of desire forms the condition for the relationship to something that exists outside the body and has to do with signifiers. In the 1970s, Lacan will name this type of *jouissance*—outside the body and related to signifiers—“phallic *jouissance*^2^,” because signification is always related to the Phallus (Φ). In *The Subversion of the Subject and the Dialectics of Desire* (Lacan, 1960b), he says that the phallic signifier is the signifier of *jouissance* and as such represents an exception in the signifying chain, because without it signifiers do not signify anything.

In the seminar *The Other Side of Psychoanalysis* (Lacan, 1969–1970) Lacan defines *jouissance* in relation to the notion of repetition. In this new conceptualization, it is *jouissance* that drives repetition, or, to put it otherwise, repetition strives for *jouissance*. Previously, the notion of repetition was related to the signifying chain and the regular return of certain signifiers. In this new version (Lacan, 1969–1970), Lacan tries to revise the notion by making use of the concept of “unary trait,” a neologism introduced in different forms as early as in his seminar on *Identification*. It is based on Frege's set theory, but also and especially on Freud's term *einziger Zug*. In *Group Psychology and the Analysis of the Ego* (Freud, 1921, p. 107), Freud speaks about this “single trait” in the context of identifying with the group leader through the ego-ideal. In the same text, he has already mentioned identification through the symptom as the second version of this single trait. The classical example is Dora's hysterical cough, a characteristic trait she copies from her father and through which she identifies with him. Lacan prefers the neologism “unary trait”, but when he speaks about it specifically with regards to *jouissance*, he says that it originates in the contingency of an encounter and instead of collectivizing, identifying the subject as in the other two cases, it characterizes him in a unique way and therefore is “distinct”. He says:

What necessitates repetition is *jouissance*, a term specifically referred to. […] As everything in the facts, in clinical experience, indicates to us, repetition is based on the return of *jouissance*. And what, in this connection, is well spelled out by Freud himself is that, in this very repetition, something is produced that is a defect, a failure” (p. 45–46). “Repetition is the precise denotation of a trait that I have uncovered for you in Freud's text as being identical with the unary trait, with the little stick, with the element of writing, the element of a trait insofar as it is the commemoration of an irruption of *jouissance*. (Lacan, 1969–1970, p. 77).

We have previously found this type of stigmata, as Soler (1991–1992) reminds us, in Freud's theory of the choice of the erotic object. Soler cites the example of the Wolfman, for whom, regardless of the reality of the primal scene—the coitus *a tergo* between his parents that he witnessed sometime between 6 months and one-and-half year of age—what remains as a memory of *jouissance* is the scene, at two-and-half years, of the governess Grusha on her knees, scrubbing the floor, and of himself looking at her from behind and urinating, which translates the child's phallic *jouissance*. According to Freud, this *jouissance* is a trait we find throughout the Wolfman's life and, as Soler (1991–1992) puts it, it functions quasi-automatically in all its different metonymical displacements. In other words, the woman “placed” in this way on the floor is one way among others of “debasing” her (a moral degradation being another option)—and from what Freud tells us, Sergei continued to feel attracted to not only maids in this position, but also to women of “loose morals.” In other words, despite Freud's efforts to give this trait, as Soler says, an Oedipal meaning through the primal scene, “in reality, it has no meaning [*sens*]; it is a trait that stigmatizes the experience of *jouissance*. So, if we want it to have a meaning, this meaning is no other but *jouissance”* (Soler, 1991–1992, lecture of 13 May 1992). Which might help make the link, in a certain way, between, in this case, the choice of the erotic object—such and such type of woman in such and such situation—and the theme of *jouissance*, in this case phallic *jouissance*, in the form of urination. It is a simple way of thinking about the unary trait as a characteristic of the experience of *jouissance*, which repeats automatically.

But repetition is not simply the repetition of the unary trait. It is also one of the consequences that the existence of the trait has for *jouissance*. According to Lacan^3^, we need three moments in the repetition of the unary trait for repetition to function. The first moment is the encounter with an experience of *jouissance*, i.e., a moment in which the unary trait erects a “monument to *jouissance*,” as he puts it. The second moment is the repetition of the trait, or rather an attempt at its repetition, because the striving for repetition results in what he calls an “immixtion of difference^4^.” In other words, what is repeated is already different, giving rise to loss as the gap between the first *jouissance* commemorated by the monument and the *jouissance* that remains after the attempt at repetition. In terms of physics, this loss is entropy^5^. The difference and loss create a supplementary “subject” to be repeated. What the third moment then repeats is then as much loss as it is the element of the *jouissance* with a difference. The unary trait introduces the dimension of repetition in two ways: as the nostalgia of loss and the search for its retrieval. In this seminar, Lacan introduces the object *petit a* as a “surplus *jouissance*” [*plus de jouir*], corresponding to Marx's notion of surplus-value [*plus value*]. The ambiguity of the term *plus* [“surplus” or “no more”] demonstrates that shutting down of *jouissance* is indispensable to the search for its “profit”: the object *petit a* commemorates the “loss of *jouissance*.” However, because it represents—as an object—the remainder of *jouissance* that has escaped the signifying process, Lacan calls it “surplus *jouissance*.”

## Language as *jouissance*

Three years later, in seminar *Encore* (Lacan, 1972–1973), it is the linguistic code itself that becomes understood as primarily *jouissance*, i.e., its communicative value becomes secondary. To name this *jouissance*, Lacan uses the term *lalangue*, a neologism he created, as he explains in his *Geneva Lecture on the Symptom* (Lacan, 1989), from the word *lallation*, which refers to the sounds emitted by a newborn before he can articulate them as speech. The signifier becomes dissociated from the signified: the first refers to *jouissance* and the second to signification. The *jouissance* of speech, of the “bla bla,” therefore acquires this dimension of the separation from the meaning of speech.

**Figure 2:**
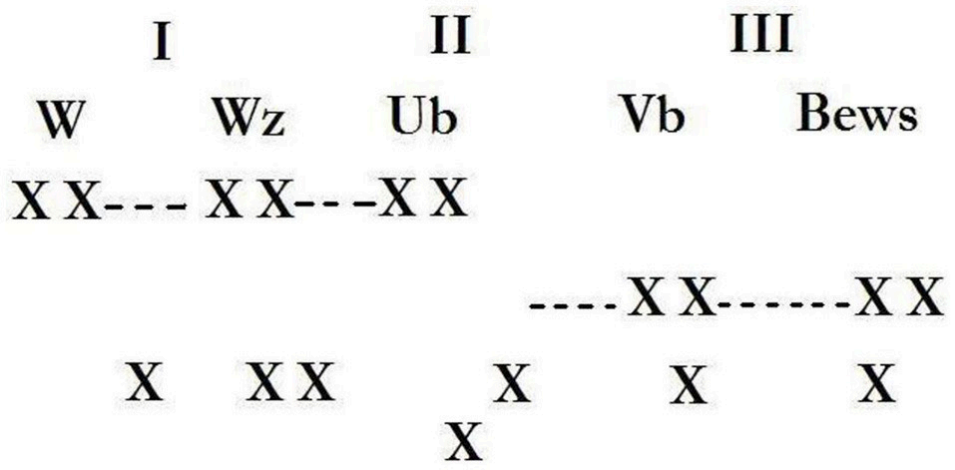
Schema from S. Freud's letter no 112 (ex-52) to W. Fliess.

According to Braunstein (2003, p. 112), we find a prefiguration of the idea of the linguistic code as *jouissance* already in Freud's letter to Fliess from 6 December, 1896^6^ (Freud, 1896b, p. 207–214). The letter outlines three consecutive systems of inscription (*Niederschriften*) of perceptions (*Wahrnehmungen*) (Figure 2). In the first system (*Wahrnehmungszeichen*), perceptions are inscribed in no specific order, as sign-traces. Following Braunstein's commentary (Braunstein, 1994, p. 176) and using the Lacanian terminology, we could say that this logical time corresponds to the mythical *jouissance*, the “*jouissance* of Being” of the first subject, which precedes the arrival of the divided subject. The first codification happens at this moment through the notion of the *trace* (rather than the signifier) inscribed in the body (or rather in the flesh, which this very process of inscription transforms into a body) which Braunstein rightly situates as what the Freud of the second topography calls, at the time of the *Mystic Writing-Pad* (Freud, 1925), the “id” (*das Id*). In this first inscription, the experiences of the Real are written simultaneously and without any (chronological or logical) order^7^. We can say that the *jouissance* of *lalangue* is the *jouissance* of Being not yet dependent on the Other's signifiers, because even though it is related to them, there is not yet any separation from this Other and, as a consequence, this Other is not “perceived” in his dimension of otherness. The second system of inscription is that of the unconscious in the first topography (*das Unbewusste*), which according to Freud is not dominated by relations of synchrony, but by other, perhaps causal, relations, which lack the linear, syntactical and logical structuring of conscious thought. However, it is already a decoding, a decryption of the signs from the previously described first moment of inscription, and it follows the primary processes of the unconscious, which, governed by the metaphorical and metonymical processes, is structured like a linguistic code. The synchrony of inscription applies here as well and, like in the precedent system, there is no contradiction and no representation of death. On the other hand, these inscriptions are linked to the Other; *jouissance* is subject to the phallic signifier and to the—non-linear—logic of “deferred action.” During the third moment of inscription, the unconscious thing-presentations of the previous moment are subject to the interpretation by the secondary processes of the preconscious, *das Vorbewusste*, and connected to word-presentations. Diachrony and temporality—in the classical sense—are applied to this system of inscriptions, which is dominated by the rules of classical logic.

Closing this short parenthesis devoted to the Lacanian elaboration of the Letter 52, let us now return to Seminar *Encore*. In this seminar, the body [*le corps*] is paramount and, as Lacan points out, homophonically contained in its very title [*En-corps*]. There is no *jouissance* outside the living body. He says: “Isn't it precisely what psychoanalytic experience presupposes?—the substance of the body, on the condition that it is defined only as that which enjoys itself” (Lacan, 1972–1973, p. 23). In this sense, there is a dissociation between *jouissance* and the big Other, an absence of relationship. The fact that there is no sexual relationship is the leitmotif of this seminar. According to Lacan, sexual *jouissance* is an impasse, because neither sex derives *jouissance* from the Other. For both, *jouissance* is mediated by the Phallus. More specifically, Lacan says: “What is known as sexual *jouissance* is marked and dominated by the impossibility of establishing as such, anywhere in the enunciable, the sole One that interests us, the One of the relation “sexual relationship”” (p. 7). He later adds: “*Jouissance, qua* sexual, is phallic—in other words, it is not related to the Other as such” (p. 9). Subsequently, when he explains the notion of the Other in the terminology of this seminar, he says that it can only be the Other sex. His formulation of Woman and the feminine *jouissance* are also key elements of this seminar: this is the Other *jouissance*, known as the *jouissance* of the mystics, who too had access to it. Hence, women have access to “phallic *jouissance*,” but their jouissance is not-all phallic, because, as Lacan says, “there is a *jouissance* that is hers, about which she herself perhaps knows nothing, if not that she experiences it” (p. 74). However, he explains that this does not happen to all women. This “Other *jouissance*” (which is not the “*jouissance* of the Other”—as Lacan has previously demonstrated, the latter is, by virtue of being the *jouissance* of the Other sex, impossible) is supplementary rather than complementary; the idea of complementarity would in fact create an illusion that the sexual relationship does exist.

**Figure 3:**
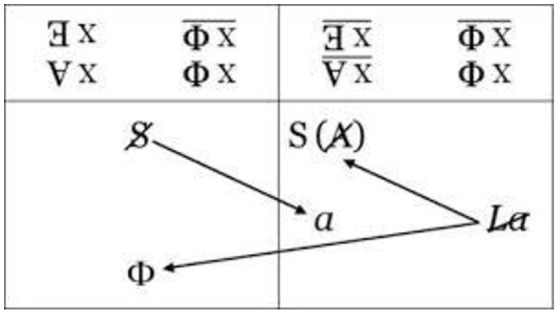
The diagram of sexual difference, J. Lacan seminar Encore.

The table of “writing” the sexual relationship—as a failure—which we find in the Seminar *Encore* (Figure 3) suggests that men fail in their attempts to relate to the Other sex, because they are in a relationship with the object *petit a* via fantasy. The body of the Other is thus reduced to the object *petit a* (written in the woman's part of the column) and all their *jouissance* is phallic *jouissance*, which contains its own limit through castration (–φ). In any case, even in the sexual act, where we could say that for the man the Phallus is momentarily embodied by the penis, the orgasm induces a refractory period that restricts jouissance; the latter remains a *jouissance* of the penis as an organ and not of the Other sex. On the other hand, for, the cause of her desire is the Phallus she does not have. This means that while in a sense she avoids the castration complex, she attaches herself to the Phallus, even if in order to do so she needs a man as her intermediary, for example by having a child.

**Figure 4:**
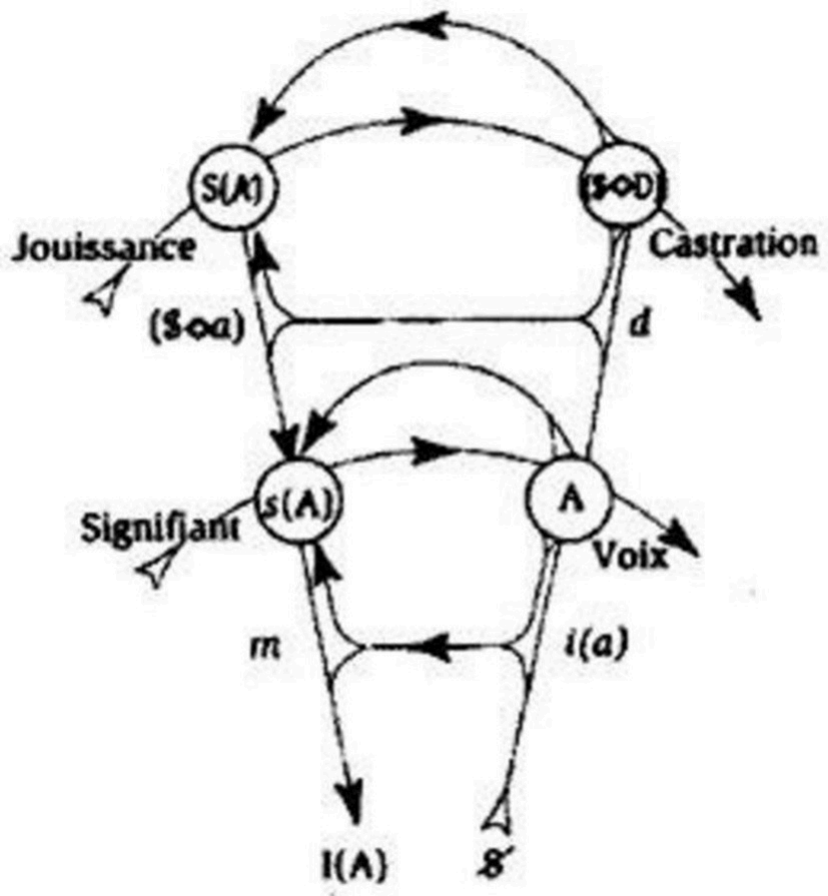
The graph of desire—complete graph, J. Lacan seminar Desir and its interpretation.

In this case, her *jouissance* is also phallic, but it leaves her the possibility of accessing the Other, “supplementary” *jouissance*. This is why she is “not-all” and is marked as barred. In the schema of the formulas, two arrows are drawn starting from the barred Woman, one toward the Phallus, which we find in the man's part of the table, the other toward the S(A), which is the symbol used in the graph of desire (Figure 4) as the signifier of the lack in the Other. This signifier represents *jouissance*, because it replaces the phallic signifier, absent from the signifying chain. As Valas (1998) points out, the *jouissance* that is excluded from the place of the Other returns in the Real, especially in the body itself. The arrow pointing toward the S(A) remains in the column that concerns Woman, who is therefore not entirely phallic. As to love—and similarly to what we have seen in the seminar on *Anxiety*—here again Lacan says that “what makes up for [*ce qui supplée*] the sexual relationship is, quite precisely, love” (Lacan, 1972–1973, p. 45). We could say that in her relationship with a man, the woman gives what she does not have by representing the Phallus (which she does not have) for a man, and this gift is love, following Lacan's definition of it, namely as “giving what one does not have.” After his reference to “feminine *jouissance*” in *Encore*, Lacan in fact never mentions it again. As a result, Lacan's belief is that the *jouissances* of the two sexes are “asymmetrical” and thus can never meet, which is another way of saying that the sexual relationship does not exist.

**Figure 5:**
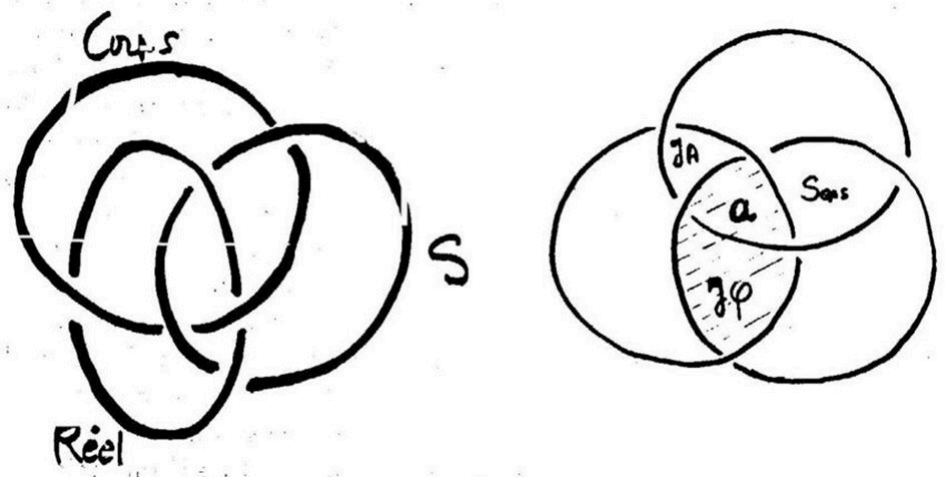
The Borromean knot, J. Lacan, conference “*La troisième*.”

We will finish with the seminar R.S.I. (Lacan, 1974–1975) and Lacan's third lecture in Rome (Lacan, 1975)—usually referred to as *La troisième—*where he makes a distinction between two types of *jouissance*: “phallic” *jouissance* and the “*jouissance* of the Other” in the sense of the objective genitive. We will not discuss this stage of his theorization of the notion of *jouissance* in great detail, because it would require an introduction to his use of the Borromean knot. We will only briefly mention that the object *petit a* is “squeezed in” (Figure 5) between:

- meaning, which forms the intersection between the Imaginary and the Symbolic;
- “phallic *jouissance*,” which we find between the Symbolic and the Real;
- the “*jouissance* of the Other” (objective genitive), which is found between the Real and the Imaginary and thus outside language. Lacan also calls it the “*jouissance* of life,” contrasting it to the “phallic *jouissance*,” which is the “*jouissance* of death,” because, as we have already said, it refers to the signifier and historicizes the subject, thus “helping” to kill, i.e., evacuate, other types of *jouissance*.

## The possible destructive effects of *jouissance* on the organism

Having followed the different stages in the development of the conceptual field of *jouissance* in Lacan's teaching, we are now going to look at the specific question of the potentially destructive effects that *jouissance* can have on the human organism, i.e., the possibility that *jouissance* produces organic deficits, especially in cases where it is not limited by the phallic function and thus regulated by language. As we will see, this will allow us to make a link between the concept of *jouissance* and certain neurophysiological notions^8^. Yet before we speak about organic deficit, let us first look at the relationship between a deficit and a flaw [*faille*]. As Lacan's teaching progresses, the essence of the human condition in fact becomes articulated to an idea of a “flaw.” In his commentary on Lacan's *Presentation on Psychical Causality* (Lacan, 1946) and critiquing theories that understand madness as a deficit, Jacques-Alain Miller argues:

A deficit can be identified in physical reality, and it is still the case that in order to treat a certain number of dysfunctions, we try to identify, via brain imaging, insufficient activity in this or that area of the brain. So these are fundamentally physical deficits. On the contrary, the flaw in question is a signifying fault, which Lacan understands as a split [*faille*] between the ego and the subject's being […] (Miller, 2008, 30 January 2008).

Lacan therefore shifts the question from the deficit to the flaw and the subject will be considered by him as a gap between signifiers; the term “rift” [*déchirement*] was already present in all his early work on psychosis (Lacan, 1938, p. 842–843) and also in his article on the *Mirror Stage* (Lacan, 1949). In the course of his teaching, this same flaw will be located on the level of the Real as impossible, on the level of the “sexual non-rapport” and in the concept of “not-all.”

The Lacanian theory, by way of its conception of the subject as a “flaw” (flaw because of the premature nature of the human being at the moment of his birth and, correlatively, because of its dependency to the Other of the language in regard to the formation of the organism itself, including the brain), allows us to clearly distinguish between the “subject” and the “brain of the person”; and this despite the tendency, in the last decades, toward the progressive cerebralization of personal identity^9^, as described by both anthropologist Vidal (2005) and sociologist Ehrenberg (2004). However, it is precisely the fact that the subject—as a flaw that enables the constitution of the human being—is separate from its brain that allows us to conceive the possibility, among other things, of the existence of psychosomatic affections, which affect the brain, and, consequently, the presence of deficiencies at that level.

Can Lacanian theory help us think about the appearance of an organic deficit? Lacan's reference to the Oedipal complex in *Presentation on Psychical Causality* (Lacan, 1946) makes it clear that what the establishment of the symbolic ternary prevents is precisely the processes of “sensitization.” He writes: “I would not hesitate to say that one could demonstrate that the Oedipal crisis has physiological echoes, and that, however purely psychological its mainspring may be, a certain “dose of Oedipus” can be considered to have the same humoral efficacy as the absorption of a desensitizing medication” (Lacan, 1946, p. 149). Much later, in his seminar *The Other Side of Psychoanalysis*, Lacan implicitly speaks about the potentially destructive power of *jouissance* over the living organism. We cite: “I have already said enough to you to know that *jouissance* is the jar of the Danaides, and that once you have started, you never know where it will end. It begins with a tickle and ends in a blaze of petrol. That's always what *jouissance* is” (Lacan, 1969–1970, p. 72). As he points out, the process of sensitization is physiological and even—if we adjust this to our current knowledge about the process—mostly neurophysiological (although there are some allergic and other physiologic mechanisms of sensitization that are not neurophysiologic); Besides, the term “sensitization” is quite similar to the term “conditioning” and according to Kandel (1991), who studied those mechanisms on aplysia, conditioning is a product of the sensitization mechanism.

## The kindling—excitotoxicity hypothesis

Taking Lacan's comments as our starting point, we can then pose the following audacious question: Can the mechanism of sensitization and its neurophysiological extensions of kindling and excitotoxicity account for the deficient phenomena affecting for example the brain^10^? In my previous work, we tried to show that the neurophysiological mechanism of *kindling*, which is related to the limbic system and has been described in psychiatry by Post (1992)^11^, initially in the context of manic-depressive psychosis, lends itself very well to such hypothesis. Post (1992) formulated the hypothesis of the neurophysiological mechanism of kindling in order to understand certain phenomena of mood disorders and other psychiatric disorders. Concerning mood disorders, he argued that a manic depressive illness can progress from a reactive mode of functioning toward an automatic mode of functioning. This happens through a series of affective episodes, which at first become reactions conditioned by specific circumstances. At a later stage, if these episodes are repeated with sufficient frequency, they become autonomous, in other words automatic. We should note that automatism depends on a state of excitation that has a tendency to self-perpetuate, hence the term “kindling.” According to Post et al., the mechanism of kindling could be linked to a certain type of genes.

Stahl (2002, from p. 385) argues that this neuronal excitation can even become toxic and destroy certain neurons. According to this author, in some clinical situations such as schizophrenia, depression, bipolar disorder, panic disorder, Alzheimer's disease, Parkinson's disease, and others, this excitotoxicity provokes neuronal apoptosis and makes these conditions irreversible, at least to some extent. Excitotoxicity is indeed a pathological process of neuronal alteration and destruction (or neurotoxicity) through the hyperactivation of glutamic acid and its analogs. However, we can see that the last author creates an amalgam between neurological (Alzheimer's and Parkinson's diseases) and psychiatric diseases (schizophrenia, depression, bipolar disorder, panic disorder), a danger we are trying to avoid by using the concept of the psychosomatic diseases of the brain. How to avoid such a confusion? We can consider psychiatric diseases as states, which are related, although not in a constant manner, to neurophysiological disorders, or even to neuropathological diseases that occur in the form of psychosomatic afflictions, which affect the brain. In the same way we consider that one intestinal disease may have a purely somatic origin whereas another might have a psychosomatic one, we can by the same token consider that some cerebral networks may be affected by a purely organic cause (neurological diseases), whereas some others, as in the case of some psychiatric disorders, may be affected by psychosomatic processes, by way of the kindling mechanism and its possible ecxitotoxicity outcome. Sometimes, the same cerebral circuits could be affected either by purely somatic causes, or by psychosomatic processes, and in that case, we can obtain resembling clinical configurations as we have previously suggested (Dimitriadis, 2013a), for example some catatonic-like states of a purely organic origin (such as the neuroleptic's malignant syndrome) which nevertheless possess some characteristics that resemble those of psychotic catatonic syndromes.

## The semiotic reduction

Beyond these questions about the neurophysiological mechanism of sensitization, the theory of psychosomatic phenomena put forth by Lacan in Seminar XI (Lacan, 1964) takes into consideration Pavlov's classical conditioning which is, according to Kandel, an elaboration of the sensitization mechanism. Specifically, Lacan argues that in cases where the signifying chain has become solidified, the dialectic of desire comes to a halt and, as a consequence, the “signifier of the desire of the Other” acquires a kind of opacity and becomes enigmatic. In this situation, it stops referring to another signifier and instead of reinitiating the subject's dialectic of desire it turns into an inductor, a signal, inducing disruptions to the needs of the soma. Thus, Lacan relates Pavlov's theory of conditioning^12^ to his own hypothesis of the solidification of the signifying chain in psychosomatic phenomena. In other words, he believes that there is an analogy between the solidified signifier and the signal of the experimenter (who rings a bell instead of showing the meat) in Pavlov's scenario, which is trying to condition a dog, a domesticated animal, i.e., one that is sensitive to the signs coming from the human other. This theorization allows us to correlate Lacan's psychoanalytic theory on psychosomatic phenomena, firstly to semiotics, and secondly to neurophysiology. As we said before, in this kind of process, the signal, thus produced, acquires an imperative “capacity” for the subject and conditions its soma; consequently, it can cause a disruption of the functions, even lesions.

When we talk about needs, we refer not only to hunger or the need for exemption. It is a matter of several homeostatic circuits of the organism that can be disturbed by desire and drive. We believe that we contribute to this question by generalizing the Lacanian theory, i.e., by positing that, by way of such a semiotic process, the brain's homeostatic circuits may be affected as well. That is to say that, in our opinion, the signal can condition not only the homeostatic circuits of the peripheral soma, but also those of the brain. We named the reduction of the signifier into the signal, or even into stimuli that self-maintain themselves, “semiotic reduction process” (Dimitriadis, 2013b,c). The tendency stimuli have to self-maintain themselves may be explained by way of the kindling mechanism we referred to before. As we will see below, the circuits that regulate our mood might be, in that regard, a preferential target.

## The semiotic reduction process and phaneroscopy

We believe that this reduction process, from the signifier to the signal or to the stimuli can be explained with the aid of Pierce's Phaneroscopy^13^ (the theory of phenomenology's categories) (Peirce, 1978), and its three categories: firstness, secondness, and thirdness. Firstness is “the mode of being of what is, as it is, positively and with no reference to anything else.” Secondness is “the mode of being of what is, as it is, in relation to a second, but without considering any third one.” Thirdness is “the mode of being of what is, as it is, by putting a second and third one in a reciprocal relationship.” Firstness relates to the immediate sentiment, secondness to reaction and to current events, and thirdness to language, law, and representation. Thirdness would be the strictly human category. The semiotics of the human being is determined by these ternary dialectics since the dialectics of his desire, processes like the co-modalization of different sensory fluxes, shared attention, play pretend, the so-called meta-representations, jokes, the structures of kinship etc. are all ternary processes. In the case of animals, it is the secondness of the signals that determine their semiotic systems.

According to ethologist Vidal (2011), “these registers of signals, of which anyone is in close relation with the stimulus it signals, to such an extent that it functions in the same way, are derived from *dyadic links* systems or from the secondness principle, whereas stimuli themselves act solely as “monads,” according to the principle of firstness.” For the signals, shifting is restricted to a relationship of synchronic presence (temporal or spatial contiguity, for example), and not to a (diachronic) relationship in reference to the absence of something, words for example. The function of language, according to Lacan (1956), is not to inform but to evoke. Therefore, the natural or conditioned reflexes, and more generally, the immediate reactions to a signal, belong to the secondness category. Stimuli do not even need another signal in order to be efficient; they act in closed-circuit (as monads) and are thus able to self-maintain themselves. With the help of Pierce's Phaneroscopy, we therefore suggest that several psychopathologies are related to a gradual transition from thirdness toward states that fall under the secondness or firstness category, a transition toward more and more automatic states.

More precisely:

– On the semiotic level, going from thirdness to secondness would mean going from the signifier to the signal, and on the clinical level, producing conditioning phenomena, and more generally, reaction phenomena. In this regard we have some classical psychosomatic phenomena like the conditioned anxiety crises, some conditioning phenomena in the case of drug addicts, reactive depressions or reactive manic states, the repetition syndrome in traumatic neuroses, certain “action-like “symptoms” etc.
– The transition from thirdness or secondness to firstness would even go beyond this reduction “stage.” We could maybe say that we go from the signifier or the signal to the stimuli themselves. In this event, we have even more automatic states, like automatic mood disorders: e.g., stable delusional mood or athymhormia in the case of schizophrenia, maniac, and depressive states that have become autonomous from their initial triggering causes, automatic states of panic, some psychosomatic phenomena that are automatic etc. In all of these situations, the signs do not come from the other, unlike conditioning where there is the signal from the other that is the triggering factor. In this case, stimuli in a way self-maintain themselves.

Needless to say that we do not maintain that there is continuity between animal and man on the basis of such an eventuality of semiotic reduction. Thirdness, even in case of these extreme situations, does persist, since it plays a constituent role for the human being, who cannot escape from it. In human beings, ternary structures subjugate (and in some way, “de-naturize”) ethological signaling systems (signals and stimuli). The fact that when thirdness is compromised as was the case in the states previously stated the human body makes itself sick, can be regarded as a strong proof of such an assertion.

## Logic of the signifier vs. logic of the sign

Independently of Pierce's Phaneroscopy, we can specify this semiotic reduction process in relation to the psychoanalytic concepts of “deferred action” and “repetition.” The symbolic, i.e., the signifiers' network of a particular subject, is not an enclosed system. Each encounter with chance may modify the string of its signifiers. Each signifier can change the whole of the signifying chain of a subject. In the case of psychoanalytic therapy, isolation of the signifier (of a padding button) may allow the subject to provide a new, retroactive meaning of his whole history. If we consider that for a given subject some signifiers have played a special role, they can be assigned different meanings during different stages of the subject's life, but they never cease being of decisive importance to the subject. However, this recurring re-determination, around the different possibilities the symbolic dictates to the subject, in the same time opens up new dimensions, on condition that the subject manages to “admit” to himself his inscription in the symbolic, i.e., that he manages to accept the limit of the castration that his personal story dictates for him. This logic is diachronic and of recurring retroaction, in the sense that the end result can influence its own cause and change it after the fact^14^. On the other hand, the logic of the signal or that of the sign is a linear logic, valid for the reflexes, be they natural or conditioned, and entails an objectification, a certain universality of reactions. This logic also entails a synchrony and/or a spatial contiguity and determines the learning process in animals.

We may therefore consider the “semiotic reduction” also in relation to its consequences regarding the termination of the “padding” by the signifying chain. As we have seen, the end of the padding may occur in various situations. The padding buttoning dictates a subjective and diachronic assumption as far as it puts in relation through the signifiers. The padding is also an assumption of contingency, of whatever new happens to the subject. According to Lanteri-Laura's expression (Lanteri-Laura, 1992), it is therefore a creative automatism^15^. When the padding buttoning stops, the encounter with the signifiers of the desire of the Other, as we stated previously with regard to psychosomatic phenomena, acquires a certain objectivity and a certain reality of presence, the latter having the force of an order, in other words the characteristics of a signal. These frozen and “imperative” signifiers are pseudo-signifiers, “cut off” from the subject's history (diachrony). They are actually signals that can trigger psychosomatic processes. This is another way of conceiving the “actual neuroses,” since the signals, unlike the signifiers, act in a synchronic and actual manner. At this stage, the *jouissance* in relation to the signification obtained in deferred action (of the mediation of the Other), that is to say the phallic *jouissance* (that has an out of body component), fades in favor of a *jouissance* of the body (of life) that is erratic and deregulated. Thus, the laws of life, in this case those of the human organism, can freely reveal themselves, instead of being subsumed and “de-naturized” by the effects of signification as before. And the physiological mechanisms (related, amongst others, to different processes like conditioning and kindling) can emerge spontaneously in the form of classical psychosomatic phenomena, various psychotic phenomena, panic attacks, addictive phenomena, post-traumatic phenomena, mood excesses, etc. More specifically, in the case of mood disorders the semiotic reduction (or the stop of the padding buttoning) might be that the affects, which are linked to the subject's signifiers—although in an indirect manner—lose that connection; they become estranged from the signifying function, thus transforming themselves into emotions (of secondness order) or into mood (of firstness order).

## The dialogue between neurosciences and psychoanalysis

Alain Vanier begins the preface of a book (Dimitriadis, 2013b) with the following paragraph:

Ansermet and Magistretti (2011) distinguish between four possible positions in order to work toward an articulation of neurosciences with psychoanalysis.

– In the first place, that of an “absolute heterogeneity” which leads to neurology absorbing part of psychiatry, since it implies an exclusive alternative between psychical “or” neurological atiology; in other words, nothing more than the old antagonism between psychogenesis and organogenesis. They observe in this respect that the question of the subject remains unresolved.
– Another approach is “superposition,” which favors an analogical model, this time without leftovers: this is the neuropsychoanalysis orientation.
– Thirdly, a “joining” model, which offers a potentially very rich perspective that, includes a certain idea of complexity.
– Finally, an approach that consists of a “crossroads of two heterogeneous orders” which is the subject of their studies (Ansermet and Magistretti, 2004, 2010b), with no hope of a “unified science,” to use Pierre Fédida's words^16^ as quoted in the publication. An interdisciplinary approach then, in a perspective where neurosciences and psychoanalysis are two very distinct fields, presenting some intersection points from which we can question their respective limits (Dimitriadis, 2013b, p. 9–10).

We criticized the first position (Dimitriadis, 2013b), which is adopted, among others, by Zénoni (1998), when he sustains the abolition of the anatomical and the biological, as far as he considers those fields irrelevant in regard to the causality of the human body's behavior—and the radical separation from animal determinism. He states for example: “There is no clash between biology and psychoanalysis because there is no conflict of jurisdiction between them. A clinical phenomenon's causality falls within the jurisdiction of either psychoanalysis or biology, it never proceeds from the interaction of the two.” We also criticized the second position (Dimitriadis, 2013b, 2015b), which can be found within the clinical-anatomical correlation method of Kaplan-Solms and Solms (2002), according to which the issue is to find anatomical connections between Freudian concepts and cerebral structures. The hypotheses presented in this paper, in regard to the concept of *jouissance*, fall under the fourth position. The intersection we have chosen is the concept of *jouissance*, in order to question the intricacy between the psychical factors and the purely organic ones—some genetic—within the development of some psychopathological phenomena, in relation to the potentially destructive effect of an excess of *jouissance* on the organism, more particularly on the brain, although destruction can reach other organs and functions, even life itself.

This type of approach seems important given the importance biological psychiatry has nowadays acquired within the field of psychiatry, whose studies are mostly about the brain, all the more so as psychotropic drugs work mainly on that level. According, for example, to Morin (2017), it is reasonable to ask ourselves if some psychoanalytical postulates, like childhood amnesia, are compatible with what is actually known about the brain. The author, a neurologist and psychoanalyst who worked by means of her psychoanalytical listening with patients with cerebral lesions, speaks in the same book of the benefit of a non-integrative approach (between neurology and psychoanalysis) in the case of some of these particular patients; an approach which, according to the author, could instruct us as to the impact of the body's image in identifications.

The approach we have chosen might contribute to a new distinction between neurology and psychiatry^17^, because today several researchers^18^ are looking forward to a new integration of the two fields. It could be of help to the study and explanation of certain pathologies which are on the fringes of neuro-psychiatry (e.g., catatoniform phenomena, athymhormia, mental confusion…^19^). It could also boost the study of biological vulnerability—genetic or epigenetic amongst others—in relation to psychopathologies. There could be for example some kind of dialogue with the genetic studies of Crow (2000, 2002) who maintains that schizophrenia is the price man has to pay for the ability to speak. If we were to follow Crow's viewpoint, language and the schizophrenic being are two sides of the same coin; Lacan (1946) affirmed the same thing (even though from a point of view quite distant from Crow's) in his address at the Bonneval Colloquium when he maintained that the man cannot be understood without insanity.

This approach could be of help for the double “therapies,” i.e., when a psychoanalytic cure is combined with the prescription of biological treatment by a medical doctor^20^, especially in defining the respective limits of those two oh so different approaches. It could also contribute to studies that look into the clinical effect of psychotropic drugs, as it can be perceived in transference^21^. This research begun in the 60's by some psychiatrists that worked also as psychoanalysts^22^, and is still ongoing^23^.

## Final remarks

Can the term “kindling” be directly referred to the notion of *jouissance*? Such a claim would of course be unjustified and we have critiqued precisely this type of amalgamation of concepts from different epistemic fields in my previous work (Dimitriadis, 2013b, 2015b). However, if we were to think about a neurophysiological mechanism that could account for the neurobiological support of *jouissance*—as a phenomenon of the living being—a mechanism of this kind (of reverse tolerance, like kindling) might be suitable. Bazan et al. (2016) have also suggested to instantiate *jouissance* physiologically, namely through the reward system of the mesolimbic pathway. This system can register a hypersensitivity for action that has previously been sealed by an experience of satisfaction and a jubilatory release of dopamine. However, the system also dissociates action from its results, which makes it structurally bound to produce derailments (the phenomenon of *autoshaping*), just like *jouissance* involves a derailment on the clinical level. In other words, it is the activation of the drive, a unique state of wanting and anticipation, that is intrinsically gratifying yet not pleasing—in the common sense of the term—and this pleasure of the drive fits very well with the notion of *jouissance*. These authors refer to the works of Robinson and Berridge (1995), who suggested that the key change in addiction had to do with a hypersensitization, via the long-term adaptations in the circuits of the mesolimbic dopaminergic system. Their theory of “incentive salience” shows the malleability of the mesolimbic dopaminergic to historical imprints, i.e., the mark through which the drive insists and commemorates at the same time. The development of an addiction would then be a concrete example of this dissociation: a situation where the drug becomes an object of pathological want even though its appreciation continues to decline. Yet the authors do not try to create an equivalence between the theoretical corpus and a physiological circuit, because psychical regularities become autonomous from physiology, even though the body imposes certain constraints on the constitution of the psyche. We should point out that while we have worked a priori independently from these authors, we have come to similar conclusions with respect to the type of physiological mechanism (sensitization) which, rather than explain can “prepare the grounds” for the types of repetitive phenomena and even neuronal destruction that we have described as the psychosomatic phenomena of the brain^24^. More specifically, we have put forth a hypothesis (Dimitriadis, 2013b,c) of the “brain's psychosomatic participation”^25^, which could potentially also be applied to psychopathological contexts other than manic-depressive psychosis (e.g., schizophrenia^26^, addictions, depression^27^, catatonia^28^, repetitive panic attacks, traumatic neurosis, and others).

Why then not presume that under certain conditions, where there is a “foreclosure of the Name-of-the-Father” or the signifying chain becomes “solidified,” the laws of the organism—such as the processes of “sensitization” and “conditioning”—can “switch on” and produce occasional deficits or even sequelae. As we have argued earlier, sensitization and conditioning could be considered as processes of “semiotic reduction.” In this same way, why not consider that certain phenomena of automatism and certain deficits (delusional moods, schizophrenic apathy, etc.) in the context of the psychoses (but not only) could be seen as psychosomatic disorders of the brain. The phenomena in question could also serve—albeit in random ways—as a kind of shield that mitigates excess *jouissance*. Calling these “deficient phenomena” might appear somewhat pejorative; however, the “psychotic prognosis” is often pejorative as well. This does not prevent such phenomena from being able to facilitate a certain stabilization, as if in these cases the de-symbolized body, i.e., the soma, “treated itself,” trying to pacify the excess of *jouissance*. In other words, if the Name-of-the-Father does not provide a “desensitizing medication” (as Lacan evoked in terms of Oedipus), the body can sometimes produce a condition that “functions” as this kind of medication via the psychosomatic *suppléance*, namely an illness of the brain that affects mood, motor skills and so on. Does this mean that in such cases we could speak about the *suppléances* of the Real of the body? In other words that, in the absence of another *suppléance* or compensation coming from the Symbolic, the Imaginary or the Real, the Real of the body self-mutilates and disconnects from the Other—and from his *jouissance*.

## Conclusions

To sum up the “types” of *jouissance* discussed above, we could say that we can distinguish three aspects of *jouissance* in Lacan's teaching: (1) a *jouissance* that is linked to signifiers, i.e., “phallic *jouissance*,” which is subject to castration and the Name-of-the-Father; (2) a *jouissance* prior to the relationship with the (non-barred) Other, one that is below [*en deçà*] the level of the signifiers and refers to the first experiences with the Other, i.e., the *jouissance* of the “Freudian Thing” that is inscribed in the body as traces (and is therefore the *jouissance* of the body), but at the same time cannot be named and therefore remains a myth. This “*jouissance* of Being” can only be deduced retrospectively, following the subject's division by the signifier, but the subject can no longer access it and it is therefore impossible. Finally, (3) a *jouissance* beyond signifiers, beyond the functioning of the Phallus and the Name-of-the-Father. This is the supplementary “Other *jouissance*,” accessible to women and mystics, which is beyond language and which speaking beings cannot articulate in words, even though women can feel it, although not all women. However, this Other *jouissance* is logically conditioned by the phallic function, even though it lies beyond this function: it exceeds language, but is not exempt from it.

Nevertheless, the *jouissance* underneath language (pre-discursive but not preverbal), the *jouissance* of the living, always remains subjacent, acting like a “parasite” toward other types of *jouissance*. It can have destructive effects on the body through physiological mechanisms that we tried to sketch out using the neurophysiological phenomenon of kindling, where the excess of excitation can produce toxicity, causing neuronal destruction. This kind of neuroplasticity, which we have described heuristically as the “psychosomatic disease of the brain” could affect, among others, brain structures related to affects, which would thus be reduced to emotions and moods. Through this same process, signifiers would be reduced, respectively, to signals, which in turn could be reduced to stimuli, with a tendency toward self-perpetuation. This would be a special mechanism of mental automatism, which could be triggered under the specific conditions of the fragility of the signifying chain (foreclosure of the Name-of-the-Father or solidification), in combination with biological factors, including genetic factors. Let us then give the last word to Freud, who writes, in *Draft K* (Freud, 1896a, p. 146): “I should say that heredity is an extra determinant in so far as it facilitates and increases the pathological affect—the determinant, that is, which in general makes possible the gradations between the normal and the extreme case. I do not believe that heredity determines the choice of the particular defensive neurosis.”

## Author contributions

The author confirms being the sole contributor of this work and approved it for publication.

### Conflict of interest statement

The author declares that the research was conducted in the absence of any commercial or financial relationships that could be construed as a potential conflict of interest. The reviewer, JF, and handling Editor declared their shared affiliation.

## Footnotes

^1^“A signifier in the Real of the analytic experience,” he says on 30 May 1967, “a new term” on 14 June 1967, the year of his unpublished seminar on *The Logic of Fantasy* (Lacan, 1966–1967).

^2^Later, in his seminar “Les non dupes errent” (Lacan, 1973–1974), he calls this type of *jouissance* “semiotic *jouissance*” (session of 11 June 1974).

^3^See Lacan's Seminar …*ou pire* (Lacan, 1971–1972) in *Autres Écrits*.

^4^“It is this act which produces, anachronically, the immixtion of the difference brought into the signifier. What it had been, when repeated, it differs, becoming a subject to repetition.” Lacan, *Compte rendu avec interpolations du Séminaire de l'Éthique* (Lacan, 1959–1960b).

^5^In his well-known book on repetition, Kierkegaard argues that repetition is always new and it is oriented toward the future rather than the past. For more details on precisely this question see Adam (2005).

^6^In the French context, this text is known as the “Letter 52”—Transl. Note.

^7^Lacan compares them to lotto tickets. See Lacan (1960a, p. 658).

^8^Jadin and Ritter (2009) argues that the *Project* is in fact a text on the neuropsychology of *jouissance*.

^9^See also Dimitriadis (2012a).

^10^Surely, the *jouissance* affects not only the brain, but the whole organism, since, as Lacan remarked (Lacan, 1972–1973), the *jouissance* is a manifestation of life. As we suggested previously, the brain is nor the subject, nor the person, despite the nowadays tendency to identify it to the latter. This is the reason, according to our hypothesis, that it could be affected—like other organs—by psychosomatic affections.

^11^Concerning the neurophysiological mechanisms of sensitization and kindling, we refer the reader to our article on Post's work (Dimitriadis, 2012b).

^12^According to Pavlov (1954), there is an intricacy between two systems of signaling in the human being: one related to the image (shared with animals), and another proper to the human language, the signals of the signals.

^13^Other authors have used Pierce's Phaneroscopy categories in the field of psychopathology (Roulot, 1992; Balat, 2000; Delion, 2000; Muller, 2000).

^14^Ansermet and Magistretti (2010a) have written about this operation, in connection to neuroplasticity.

^15^See also Dimitriadis (in press).

^16^“where comparative epistemology of the models and their critical theoretical functioning would play the deciding role into realizing their operative limits and their aptitude to be transformed reciprocally; in which case, the aim would be a mutual communication rather than the aspiration toward a *unified science*,” Fédida (1992), p. 295.

^17^In France, these two specializations are considered distinct since 1968.

^18^See, for instance, Price et al. (2000).

^19^See Dimitriadis (2013a).

^20^See Dimitriadis (2014).

^21^Incidentally, the prescription of anti-epileptic drugs in the case of mood disorders has increased firstly because of some observations by Lambert et al. (1966) about Depamide®, and secondly in connection with Post's research concerning the kindling mechanism, which was initially conceived by Goddard (1981), in relation to epilepsy.

^22^See, for instance, Conté (1963), and Green (1961).

^23^See, for instance, Kapsambelis (1994) and Dimitriadis (2014).

^24^It is perhaps also not a coincidence that among the first psychoanalytic works on the search for neuronal mechanisms—that could be related to certain psychic processes—we find Lawrence Kubie's work (Kubie, 1941) on the repetition in neurosis. According to some authors (for example, Le Roux, 2007), Lacan was familiar with Kubie's theory of “reverberating closed circuits.”

^25^Silvano Arieti discussed this possibility as early as in the 1960s. For a review of the literature on the prehistory and history of this hypothesis, see Dimitriadis (2012a).

^26^See Dimitriadis (2013b,c, 2015a).

^27^See Dimitriadis (2014).

^28^See Dimitriadis (2013a).
